# P-346. Implementation of an Inpatient HIV Antiretroviral Transitions of Care Pilot at a Veterans Affairs (VA) Medical Center

**DOI:** 10.1093/ofid/ofaf695.564

**Published:** 2026-01-11

**Authors:** Clayton B Nance, Pawlose Ketema, Amy Weintrob, Rachel V Denyer, Daniel Seeger, Helena Holmgren

**Affiliations:** Washington D.C. VA Medical Center, Washington, DC; Veterans Affairs VA National Capitol Care Network VISN5 Antimicrobial Stewardship Clinical Collaborative of VA Medical Centers in Washington DC/Martinsburg WV/Baltimore MD/Clarksburg WV/Beckley WV/Huntington WV, Washington, District of Columbia; Washington DC VA Medical Center/ George Washington University, Washington, District of Columbia; Washington DC VA Medical Center/ George Washington University, Washington, District of Columbia; Washington D.C. VA Medical Center, Washington, DC; George Washington University/Washington D.C. VA Medical Center, Washington, District of Columbia

## Abstract

**Background:**

During transitions of care (TOC), people living with HIV (PLWH) often warrant additional attention beyond a routine medication reconciliation. In August 2024, a single center pharmacist-led quality improvement pilot program was begun to enhance the care of this high-risk population during new hospital admissions. An aim of this pilot program was to assess the impact of inpatient HIV TOC on HIV treatment and linkage to care.

**Figure:**
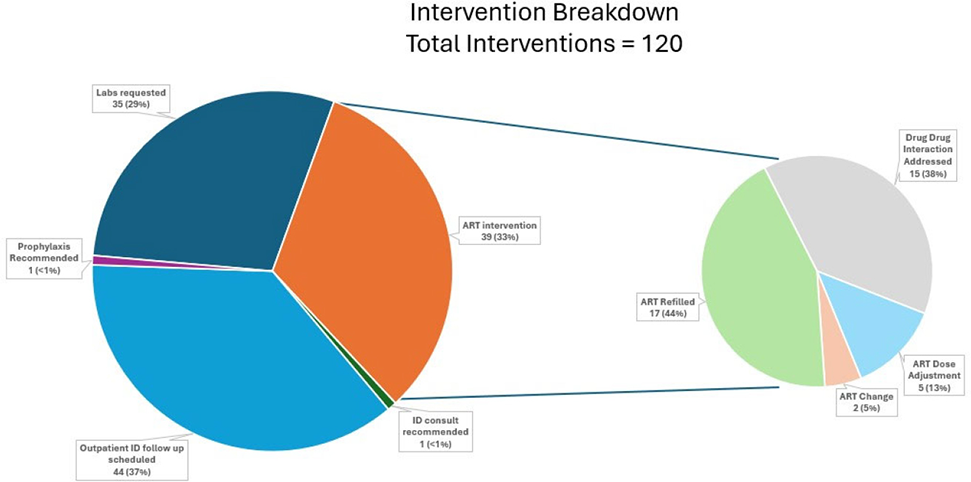

**Methods:**

Between August 1^st^, 2024, and March 31^st^, 2025, electronic medical record (EMR) alerts for inpatient antiretroviral therapy (ART) orders were reviewed Monday through Friday by the Infectious Diseases (ID) pharmacist. Patients were only reviewed once per admission and were excluded if a formal ID consult had been placed. The ART review included HIV treatment history, medication interactions, and established linkage to HIV care if needed. These findings were documented as an HIV pharmacotherapy note in the EMR. Interventions were made by the ID clinical pharmacist and/or in collaboration with an ID physician or the inpatient medical team.

**Results:**

During the 8-month pilot period, there were 92 ART reviews (70 unique patients) of which 67 resulted in at least 1 intervention (73%). There were 120 total interventions made. Routine HIV laboratory monitoring recommendations were made to primary teams in 35 cases. ART interventions were made in 39 cases including: addressing drug-drug interactions (15), dose adjustment (5), ART refills (17), and ART changes (2). Outpatient ID clinic follow up was arranged in 44 cases for 38 unique patients. Of these, 20 subsequently attended a follow up appointment including 3 patients who had previously been out of care.

**Conclusion:**

HIV ART monitoring at transitions of care resulted in tangible benefits such as improved medication safety and linkage to care for PLWH at our medical center. A simple EMR alert resulted in a specialty pharmacist review that was shown to be feasible and resulted in multiple episodes of ART optimization and improved patient care. This inpatient program will continue at our medical center and is expected to lead to improvements in health outcomes.

**Disclosures:**

All Authors: No reported disclosures

